# Transcriptional consequences of impaired immune cell responses induced by cystic fibrosis plasma characterized via dual RNA sequencing

**DOI:** 10.1186/s12920-019-0529-0

**Published:** 2019-05-22

**Authors:** Justin E. Ideozu, Vittobai Rangaraj, Hiam Abdala-Valencia, Xi Zhang, Manoj Kandpal, Marc A. Sala, Ramana V. Davuluri, Hara Levy

**Affiliations:** 1 Division of Pulmonary Medicine, Ann & Robert H. Lurie Children’s Hospital of Chicago, Chicago, IL 60611 USA; 2 Human Molecular Genetics Program, Stanley Manne Children’s Research Institute, Chicago, IL 60614 USA; 3 Northwestern University Feinberg School of Medicine, Chicago, IL 60611 USA

**Keywords:** Cystic fibrosis, miRNA, Immune response, PBMCs, RNA-Seq

## Abstract

**Background:**

In cystic fibrosis (CF), impaired immune cell responses, driven by the dysfunctional CF transmembrane conductance regulator (*CFTR*) gene, may determine the disease severity but clinical heterogeneity remains a major therapeutic challenge. The characterization of molecular mechanisms underlying impaired immune responses in CF may reveal novel targets with therapeutic potential. Therefore, we utilized simultaneous RNA sequencing targeted at identifying differentially expressed genes, transcripts, and miRNAs that characterize impaired immune responses triggered by CF and its phenotypes.

**Methods:**

Peripheral blood mononuclear cells (PBMCs) extracted from a healthy donor were stimulated with plasma from CF patients (*n* = 9) and healthy controls (*n* = 3). The PBMCs were cultured (1 × 10^5^ cells/well) for 9 h at 37 ° C in 5% CO_2_. After culture, total RNA was extracted from each sample and used for simultaneous total RNA and miRNA sequencing.

**Results:**

Analysis of expression signatures from peripheral blood mononuclear cells induced by plasma of CF patients and healthy controls identified 151 genes, 154 individual transcripts, and 41 miRNAs differentially expressed in CF compared to HC while the expression signatures of 285 genes, 241 individual transcripts, and seven miRNAs differed due to CF phenotypes. Top immune pathways influenced by CF included agranulocyte adhesion, diapedesis signaling, and IL17 signaling, while those influenced by CF phenotypes included natural killer cell signaling and PI3K signaling in B lymphocytes. Upstream regulator analysis indicated dysregulation of CCL5, NF-κB and IL1A due to CF while dysregulation of TREM1 and TP53 regulators were associated with CF phenotype. Five miRNAs showed inverse expression patterns with three target genes relevant in CF-associated impaired immune pathways while two miRNAs showed inverse expression patterns with two target genes relevant to a dysregulated immune pathway associated with CF phenotypes.

**Conclusions:**

Our results indicate that miRNAs and individual transcript variants are relevant molecular targets contributing to impaired immune cell responses in CF.

**Electronic supplementary material:**

The online version of this article (10.1186/s12920-019-0529-0) contains supplementary material, which is available to authorized users.

## Background

In cystic fibrosis (CF), the lack of a functioning CFTR protein, due to *CFTR* gene variants, results in increased susceptibility to lung infections and pancreatic insufficiency [1]. Chronic progressive lung disease due to colonization with *Pseudomonas aeruginosa* (*Pa*) infection is the chief cause of CF morbidity and mortality [2, 3]. However, the relationship between genotype and phenotype is complex, with several clinical characteristics caused by various combinations of *CFTR* variants [4, 5]. Although the genetic characterization of patients has been greatly improved by next-generation sequencing approaches [6–8], their genetic and clinical heterogeneity remains a major therapeutic challenge [9]. The characterization of molecular mechanisms underlying CF pathology is, therefore, a critical step to identifying novel molecular targets with therapeutic potential in CF.

In attempts to understand the mechanisms underlying how dysfunctional CFTR leads to increased susceptibility to chronic lung infections, most studies investigate CF epithelial cells [10]. However, several studies have shown that impaired immune cell responses are central to the lung disease severity in CF [2, 11, 12], which indicates that both epithelial and immune cells are relevant players involved in CF pathology. As in other diseases [13], the CF host immune system can respond to pathogens by triggering the expression of genes, their isoforms, and their regulators. These expression features can be assessed using advanced high-throughput transcriptomic technologies, and this has already led to the identification of some dysregulated immunity-related genes in CF epithelia [14, 15] and blood cells [16]. Peripheral blood mononuclear cells (PBMCs) can respond to extrinsic stimuli and can be used as effective model systems for investigating immune cell responses in many diseases [17, 18]. By utilizing microarrays to profile transcriptional signatures of PBMCs stimulated with CF plasma, it was reported that several dysregulated immunity-related genes characterized CF and its phenotypes [19, 20]. Although specific findings vary between previous studies, dysregulation or imbalances of immune molecules are now considered dominant features in CF [10, 21, 22]. However, it remains poorly understood what drives the observed differences in expression signatures of immune molecules.

Alternative splicing is one such biological mechanism through which gene expression is controlled, and most genes have multiple transcript variants (isoforms) that can have different functions in different cell-types or disease states [23, 24]. Alternative splicing is profoundly prevalent in the immune cells, where it dictates the function of many signaling molecules [25]. Several individual transcripts from multiple-transcript genes have been associated with many diseases [24, 26, 27], but it remains unclear whether certain individual transcript variants can characterize CF and its phenotypes. In addition, considering that noncoding RNAs such as microRNAs (miRNAs) are known to regulate the expression of their genes and their altered expression has been implicated in a variety of human diseases, including CF [28–31], miRNAs may be involved with regulating key dysregulated immunity-related genes in CF.

RNA Sequencing (RNA-Seq) has emerged as a powerful high-throughput technology that allows for efficient and accurate quantification of genes, transcripts, and non-coding RNAs such as miRNAs in the transcriptome [32]. When used in combination with in silico functional genomics approaches, complex mechanisms underlying the pathogenesis of several diseases can be unraveled [33–35]. We performed dual RNA-Seq using plasma-stimulated PBMCs followed by functional genomics to identify differentially expressed genes, transcript variants, and miRNAs that characterize impaired immune responses influenced by CF and its phenotypes. We identified several dysregulated genes, transcripts, and miRNAs potentially relevant to dysregulated immune processes that characterize CF and its phenotypes. Confirmatory studies are needed to validate specific findings.

## Methods

### Study population

**Fig. 1:**
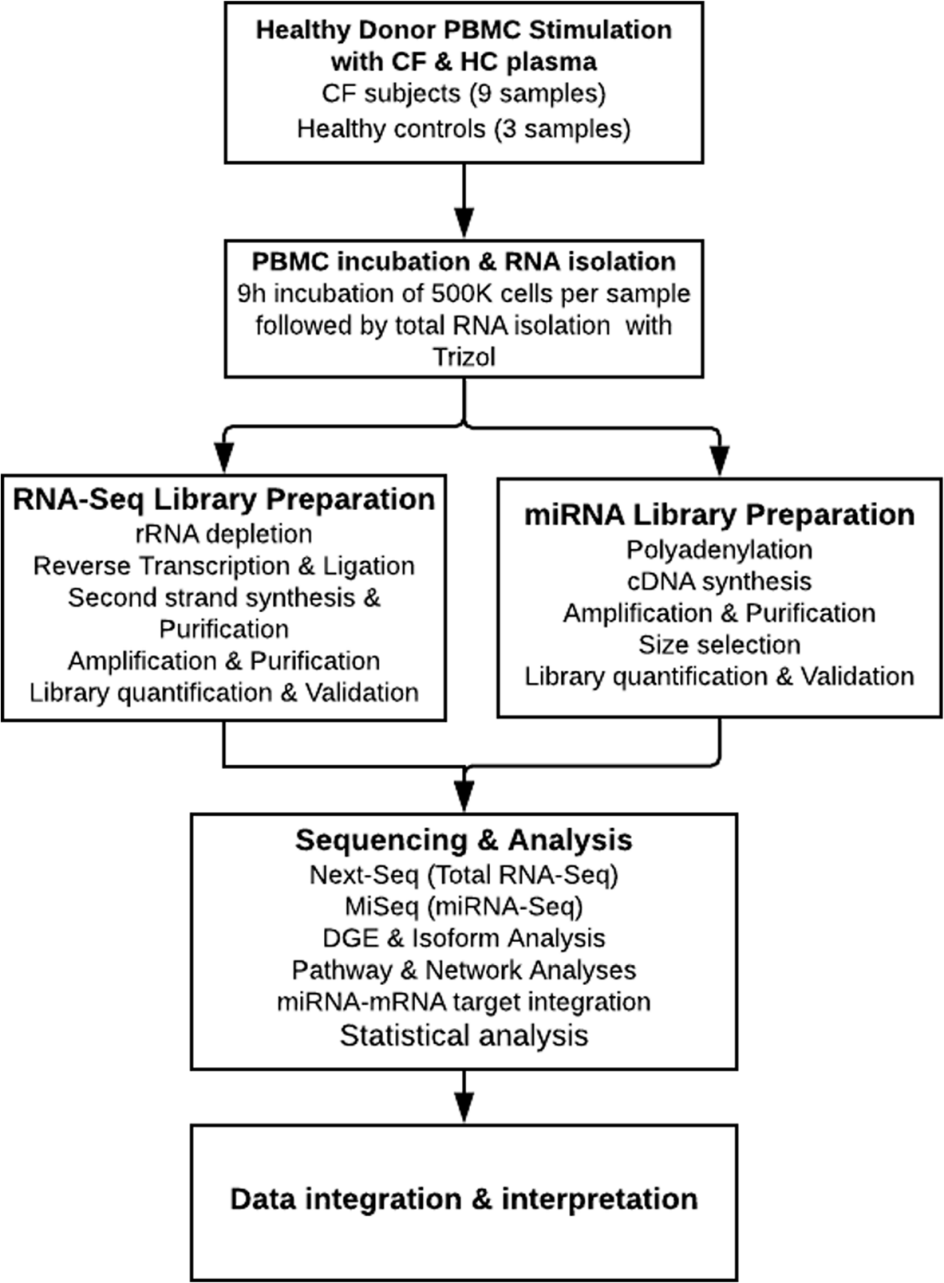
Illustration of the workflow. Healthy donor PBMCs, stimulated with plasma from 9 CF patients and 3 HC, were the source of RNA for transcriptome sequencing. The CF subjects were diagnosed based on *CFTR* genotype and sweat chloride test using published guidelines [36, 37]. Following culture, the extracted total RNA from each sample was processed for simultaneous total RNA-Seq and miRNA-Seq. RNA-Seq was performed using the Illumina Next-Seq instrument at the RNA-Seq Center, Division of Pulmonary and Critical Care, Northwestern University, while miRNA-Seq was performed in-house using an Illumina MiSeq instrument. The generated expression signatures were analyzed to identify CF-relevant molecules while patient biochemical, clinical, and genetic variables were utilized for statistical analyses

A total of 9 CF and 3 healthy control (HC) subjects were recruited at the Children’s Hospital of Wisconsin (Milwaukee, WI, USA) and the Ann & Robert H. Lurie Children's Hospital of Chicago (Chicago, IL, USA). The study was approved by the Institutional Review Boards (IRB# CHW 07/72, CTSI 847, 2015-400) and written informed consent was obtained from the subjects, their parents, or legal guardians. For each sample, peripheral blood was drawn into citrate dextrose solution A or K^+^ ethylenediaminetetraacetic acid (EDTA) anticoagulant and plasma isolated using Ficoll Histopaque (Sigma-Aldrich Corporation, MO, USA). Plasma was then stored at − 80 **°**C until needed for further processing. All CF subjects were diagnosed based on results of sweat chloride test and *CFTR* genotype, using published guidelines [36, 37]. The sweat chloride level is an important biochemical variable known to be significantly elevated in CF patients with more severe disease [38]. Other relevant clinical variables such as pancreatic function status, mucoid *Pa* infection status, and the forced expiratory volume in 1 s (FEV_1_) percent predicted were recorded for each CF patient at the time of sample collection. Mucoid *Pa* infection was reported as positive microbiological growth from sputum culture detected during the time of enrollment while mucoid *Pa* negative was reported as negative for mucoid *Pa* within 6 months pre/post sample enrollment. Based on pancreatic status: CF subjects carrying pathogenic variants (class I, II, and III) and/or positive for fecal elastase test (< 200 μg/g) were diagnosed as pancreatic insufficient (PI) and assigned to the Severe disease group, while those with one mild variant (Class IV and V) and pancreatic sufficiency (PS) were assigned to the Mild group, as previously defined [39–42]. We simultaneously performed total RNA and miRNA sequencing to identify plasma-induced signatures of PBMCs that differ in expression levels due to CF (by comparing 9 CF subjects vs 3 HC) and those that differ due to CF phenotypes (by comparing between PI/Severe (*n* = 6) and PS/Mild (*n* = 3) CF subjects) (Fig. 1).

### PBMC culture and RNA isolation

Cryopreserved PBMCs from a well-characterized healthy Caucasian HLA-A2 male donor (UPN727) were acquired from Cellular Technology Limited (CTL, OH, USA), washed, and thawed according to the vendor’s recommendation. The PBMCs were then cultured (1 × 10^5^ cells/well) for 9 h at 37 ° C in 5% CO_2_ with 20% plasma isolated from CF subjects and HC, as previously described [19]. Following culture, total RNA was extracted using TRIzol Reagent (Invitrogen Life Technologies, MA, USA) and RNA integrity quantified in Bioanalyzer 2100 using the Agilent RNA 6000 Nano kit (Agilent Technologies, CA, USA).

### Library preparation and sequencing

Total RNA-Seq and miRNA-Seq libraries were prepared simultaneously for each of the 12 samples (9 CF and 3 HC) using the same total RNA source. For total RNA-Seq, ribosomal RNA (rRNA) was first depleted from total RNA (26 ng) using RiboCop rRNA depletion kit (Lexogen, Vienna, Austria) prior to strand-specific single-ended library preparation using Sense Total RNA Library Prep Kit (Lexogen, Vienna, Austria). Briefly, the rRNA-depleted RNA were hybridized to heterodimers containing Illumina-compatible linker sequences. Following reverse transcription and ligation, end-repaired cDNA fragments were then generated. The double-stranded cDNA libraries generated during the second strand synthesis were then purified, amplified in the GeneAmp PCR System 9700 (Applied Biosystems, CA, USA), and finally purified for sequencing. Libraries were sent to the RNA-Seq Centre, Division of Pulmonary Critical Care at Northwestern University for sequencing (76 bp read length) using a Next-Seq sequencer (Illumina, CA, USA).

For miRNA-Seq, libraries were generated using the SMARTer smRNA-Seq kit (Takara Bio, CA, USA), according to the vendor’s recommended protocols. Briefly, total RNA (7 ng) was first polyadenylated to facilitate oligo (dT)-primed cDNA synthesis. Amplification of cDNA and addition of full-length Illumina adapters was performed using GeneAmp PCR System 9700 (Applied Biosystems, CA, USA). The PCR products were purified using the NucleoSpin Gel and PCR Clean-Up kit (Macherey–Nagel, Düren, Germany). Purified libraries were quantified using Qubit 3.0 Fluorimeter (Thermofisher Scientific, MA, USA), and size-selected using Agencourt AMPure XP Beads (Beckman Coulter, CA, USA). The multiplexed single-ended purified libraries were sequenced using a MiSeq sequencer (Illumina, CA, USA).

### Data processing and analysis

The raw sequencing reads were assessed, processed, and analyzed using Partek® Flow (Partek, MO, USA). For total RNA-Seq, the first nine non-specific bases introduced by library chemistry were trimmed from all reads. Bases called at less than 99.9% accuracy (Q < 30) were filtered out and the high-quality sequencing reads aligned to the human reference genome (hg19) using STAR aligner (v2.4.1d). The Partek E/M algorithm, using RefSeq Transcript 81 as the annotation model, was used for quantifying gene and transcript features. All low-expression features with ≤10 reads were filtered out from the quantification results. In order to minimize the impact of possible sources of systematic variation, such as sequencing depth, gene length, and composition, feature counts were normalized using Total Count algorithm and differential expression estimated using the Limma Voom method [43]. Molecule type for each of the normalized expression features was annotated with the Ingenuity Pathway Analysis (IPA) tool (Qiagen, CA, USA) using default settings and the results were displayed in a pie chart. Differential expression analysis of features was performed at the gene-level (testing the differences in the overall transcriptional output of the quantified genes between the conditions) and at the transcript-level (testing the differences in expression of each individual transcript between the conditions), as previously described [44]. Differentially expressed signatures meeting a significance threshold of false-discovery rate (FDR) < 0.1 (ANOVA F-test, *p* < 0.005) with at least 2-fold change (FC) difference were considered for further functional analysis.

For small RNA sequencing reads, we filtered all low-quality reads (Q < 30) and trimmed the first 3 nucleotides of all reads inserted due to the chemistry of the SMARTer smRNA-Seq kit (Takara Bio, CA, USA). Adapter sequences were identified in all reads and trimmed from the 3-prime end. Reads shorter than 15 nucleotides were discarded after trimming and Bowtie (v1.0.0) was used to align the remaining high quality reads to the human reference genome (hg19) using miRBase mature miRNAs (v21) annotation [45]. The Partek Quantify to Annotation model, with a minimum feature-read overlap of 100%, was then used to estimate miRNA abundance. The trimmed mean of M values (TMM) algorithm, demonstrated to be effective in minimizing variance when normalizing low abundance RNA species such as miRNAs without introducing noise [46], was used to normalize the miRNA counts. Differential miRNA expression was then estimated using the Limma Voom method [43]. Low expressed miRNAs with less than 10 reads in at least 50% of samples were filtered out and only those meeting a significance threshold (ANOVA F-test, FDR < 0.05, log2 FC ≥ 2) were considered as differentially expressed and prioritized for functional analysis in this study.

The normalized counts were used for displaying results in MA-plots, principal component analysis (PCA) graphs, and volcano plots.

### Functional analysis

Gene ontology (GO) enrichment, biological pathway, and upstream analyses were performed using the differentially expressed genes identified from the two comparisons (CF vs HC; Severe vs Mild CF). The enrichment analyses were performed using PANTHER (v13.1) [47] to identify over-represented GO categories (biological processes, molecular functions, and cellular components). The categories were then ranked based on the enrichment score, which was deduced using the negative natural logarithm of the *p*-values. An enrichment score of 3 corresponds to a significance threshold of *p* < 0.05. The top 10 most enriched categories were identified in both comparisons (CF vs HC; Severe vs Mild CF). Functional analysis was further performed with the IPA tool (Qiagen, CA, USA) to identify dysregulated canonical immune pathways and predict potential upstream regulators influencing the expression of the dysregulated genes. An absolute value of z-score > ±2 (Fisher’s Exact test, *p* < 0.05) was considered statistically significant for prediction of effect. Furthermore, the Isoprofiler tool in IPA (Qiagen, CA, USA) was utilized to functionally characterize all transcripts captured by RNA sequencing. We characterized relevant protein-coding isoforms differentially expressed and explored their association with disease processes and some of the predicted dysregulated pathways. The results of the functional analyses were displayed as bar charts or network graphs.

### miRNA target prediction

The interaction between differentially expressed miRNAs and mRNAs were analyzed in IPA using the miRNA Target Filter tool (Qiagen, CA, USA). Predicted mRNA targets for differentially expressed miRNAs were identified based on four algorithms (TargetScan, TarBase, miRecords, and Ingenuity Knowledge Base). The miRNA-mRNA pairs in the experimental datasets with inverse expression correlation and relevance to immune processes/pathways listed in the IPA knowledge base were then characterized and displayed as networks.

### Statistical analysis

Statistical analyses were conducted using SPSS 25.0 software (IBM, USA). Normality tests for continuous variables were performed with a Shapiro-Wilk test. Data were presented as median and interquartile range (IQR). As appropriate, a *t-*test was performed for comparison between two groups (CF vs HC; Severe vs Mild). Categorical variables were tested for association with Chi-square or Fisher’s exact test. Differences with *p < 0.05 were considered as statistically significant.

## Results

### Baseline characteristics of study samples

Simultaneous total RNA-Seq and small RNA-Seq was performed in 9 CF samples and 3 HC samples to identify differentially expressed plasma-induced signatures. Clinical and demographic information for the samples are presented in Table 1. Among the 9 CF subjects, 6 were assigned to the Severe phenotype group while 3 were assigned to the Mild group as described in methods. The median (interquartile range [IQR]) age for those in the Severe group was 10 (7, 25) years and 6 (5, 7) years for those in the Mild group. Males accounted for 33% in both Severe and Mild phenotype groups. The median (IQR) sweat chloride level was 108 (100, 125) mmol/L in the Severe CF group, which was significantly (*p* = 0.009) higher than 67 (23, 77) mmol/L observed in the Mild CF group. About 67% of CF patients in the Severe group were positive for mucoid *Pa* while those in the Mild group were all negative for the lung infection. The median FEV_1_ percent predicted was 102 (85, 113) for the Severe group and 113 (110, 118) for the Mild group. Compared to the HC samples, the median (IQR) age of the CF patients recruited in this study was 7 (6, 18) years while the HC samples had a median (IQR) age of 8 (8, 8) years. Again, males accounted for 33.3% in both CF subjects and HC samples.

**Table 1:** Demographic, clinical and genetic information for study cohort

Comparison	Parameter	Group1	Group2	*p-value*
1	Condition	CF (*n* = 9)	Healthy controls (*n* = 3)	NA
	Age in years, median (IQR)	7 (6, 18)	8 (8, 8)	NS
	Gender: Male, n (%)	3 (33.3%)	1 (33.3%)	NS
2	CF Phenotype	Severe^1^ (*n* = 6)	Mild^2^ (*n* = 3)	NA
	Age in years, median (IQR)	10 (7, 25)	6 (5, 7)	NS
	Gender: Male, n (%)	3 (33.3%)	1 (33.3%)	NS
	Gender: Male, n (%)	2 (33.3%)	1 (33.3%)	NS
	Sweat chloride, median (IQR)	105 (100, 123)	67 (30, 77)	0.007*
	Mucoid *P. aeruginosa*, n (%)	4 (66.7%)	0	NS
	FEV_1_% predicted, median (IQR)	103 (85, 113)	113 (110, 118)	NS
	F508del homozygotes, n (%)	6 (66.7%)	0	0.01^†^

*T-test. ^†^Fisher’s exact test. ^1^The severe group are homozygous for F508del (c.1521_1523delCTT), a class II *CFTR* variant associated with pancreatic insufficiency (PI) and more severe disease. ^2^The mild group were pancreatic sufficient and have a combination of F508del (c.1521_1523delCTT) and either S1251 N (c.3752G > A) and R117H;7 T (c.350G > A;1210 − 12 T [7]), and EX-19dup. The severity status was assigned as previously described [39–42]. NA not applicable. NS not significant

### Total RNA-Seq analysis

Total RNA-Seq reads were generated from PBMCs stimulated with plasma from CF subjects and healthy controls. The reads were aligned to the human reference genome using a splice-junction aware aligner (Star v2.4.1d) and an average of 36 million reads was generated for each sample with an average Phred quality score of 31.2. A high percentage (87%) of these reads aligned to the human reference genome (hg19) (Additional file 1: Table S1). Quantification of the high-quality reads using human reference transcriptome annotation (RefSeq Transcripts 81) resulted in estimates of expression levels for 27,523 genes, corresponding to 64,886 transcripts, for all CF and HC samples. After filtering low expressed features (≤10 reads), 15,257 genes, corresponding to 32,399 transcripts, were retained for normalization and differential expression analysis at gene and transcript level.

### CF plasma-induced characteristic gene expression signatures in non-CF PBMCs

**Fig. 2:**
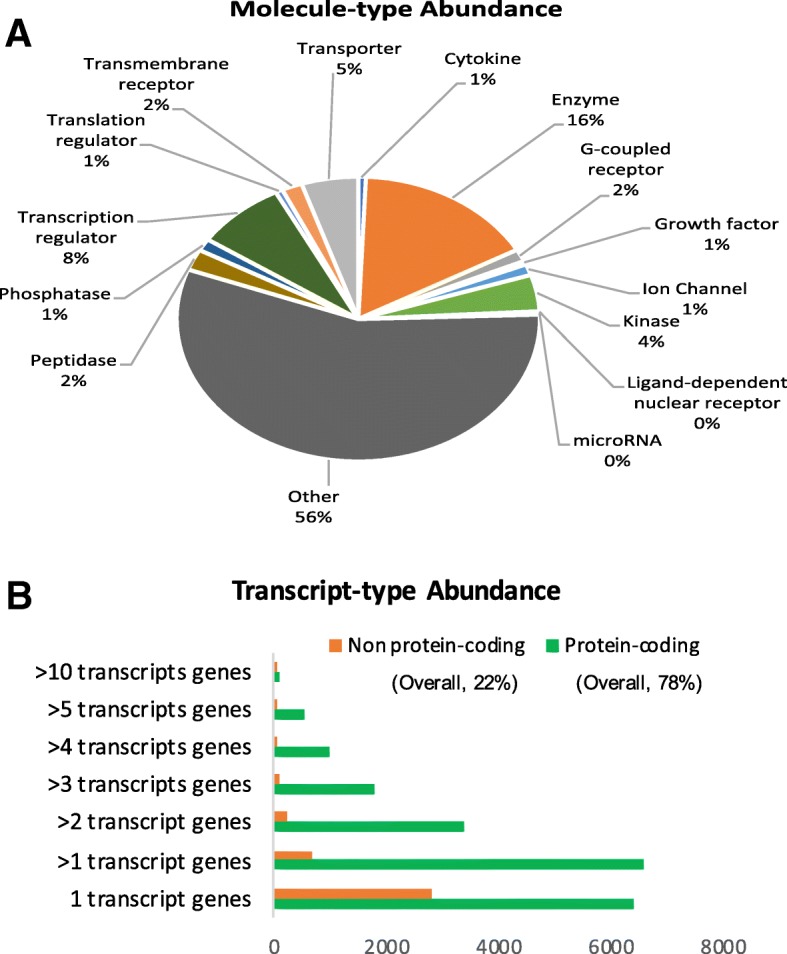
Diverse range of transcripts and genes were abundant in datasets. **a** Genes encoding a diverse range of molecules were detected. **b** Profiling of transcript-type abundance in expression datasets after filtering for low-expression transcripts with ≤10 reads showed that the majority of transcripts expressed in plasma-stimulated PBMCs were protein-coding (78%) and both single and multiple-transcript genes were of high abundance in the expression dataset

**Fig. 3:**
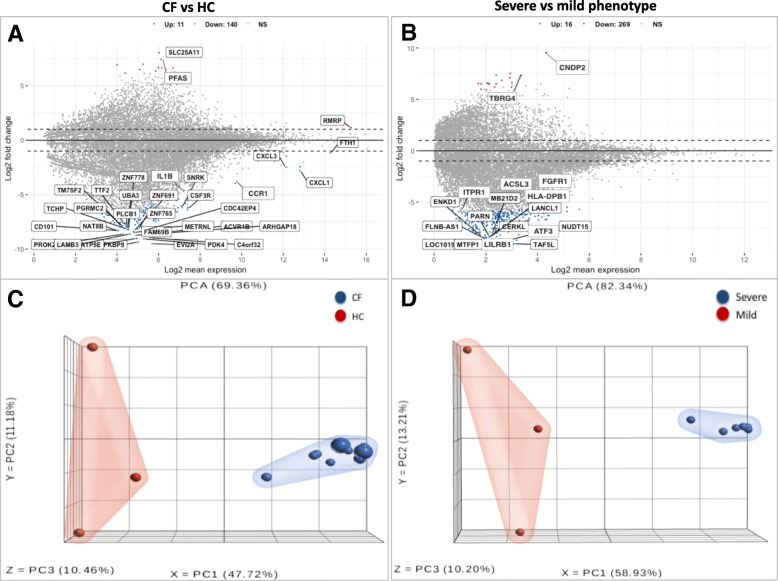
Gene expression signatures differ based on CF disease and its phenotypes. **a** & **b** MA-plot of total RNA-Seq data with Log2-transformed fold change values (y-axis) plotted against Log2-transformed mean gene expression values (x-axis). Upregulated genes are represented in red, downregulated in blue, and those not meeting the threshold (*P* < 0.05, FDR < 0.1, > ± 2 FC) in grey (NS). One hundred and fifty-one genes were differentially expressed when comparing CF and HC (**a**). There were 285 genes differentially expressed based on CF phenotype (**b**). **c** & **d** PCA graph of differentially expressed genes. Each dot represents a study sample assigned to one of the experimental groups, which are highlighted in either blue or red. The three principal components (X, Y, and Z) explain 69% of the expression variance plotted for the 12 samples in CF compared to HC group (**c**), and 82% of the expression variance due to CF phenotype (**d**), respectively

Genes encoding a diverse range of molecules were detected in the dataset following transcriptome analysis of the non-CF PBMCs induced by plasma from CF and HC subjects (Fig. 2 a). We identified 151 plasma-induced gene expression signatures differentially expressed between CF and HC subjects (FDR < 0.1, *p* < 0.005, > ±2 FC). Among these, 140 genes (93%) were downregulated while 11 genes (7%) were upregulated in CF compared to HC (Fig. 3 a, Additional file 1: Table S2). Amongst the top abundantly expressed genes that were dysregulated, *CSF3R*, *CXCL1*, *CXCL3*, *IL1B,* and *FTH1* are associated with several immune pathways. To better characterize the transcriptional repertoire of CF subjects based on phenotypes, we then compared differential gene expression levels between those with severe and mild phenotypes. Our results showed 285 genes were significantly (FDR < 0.1, *p* < 0.005, > ± 2 FC) differentially expressed between the two groups. Among these, CF subjects in the Severe group had 269 downregulated genes (94%) compared to those in the Mild group (Fig. 3 b: Additional file 1: Table S3). PCA using the top varying plasma-induced gene expression signatures segregated CF from HC subjects (Fig. 3 c) and Severe from Mild CF phenotypes (Fig. 3 d).

### Individual transcript variants expression levels differ in CF

**Fig. 4:**
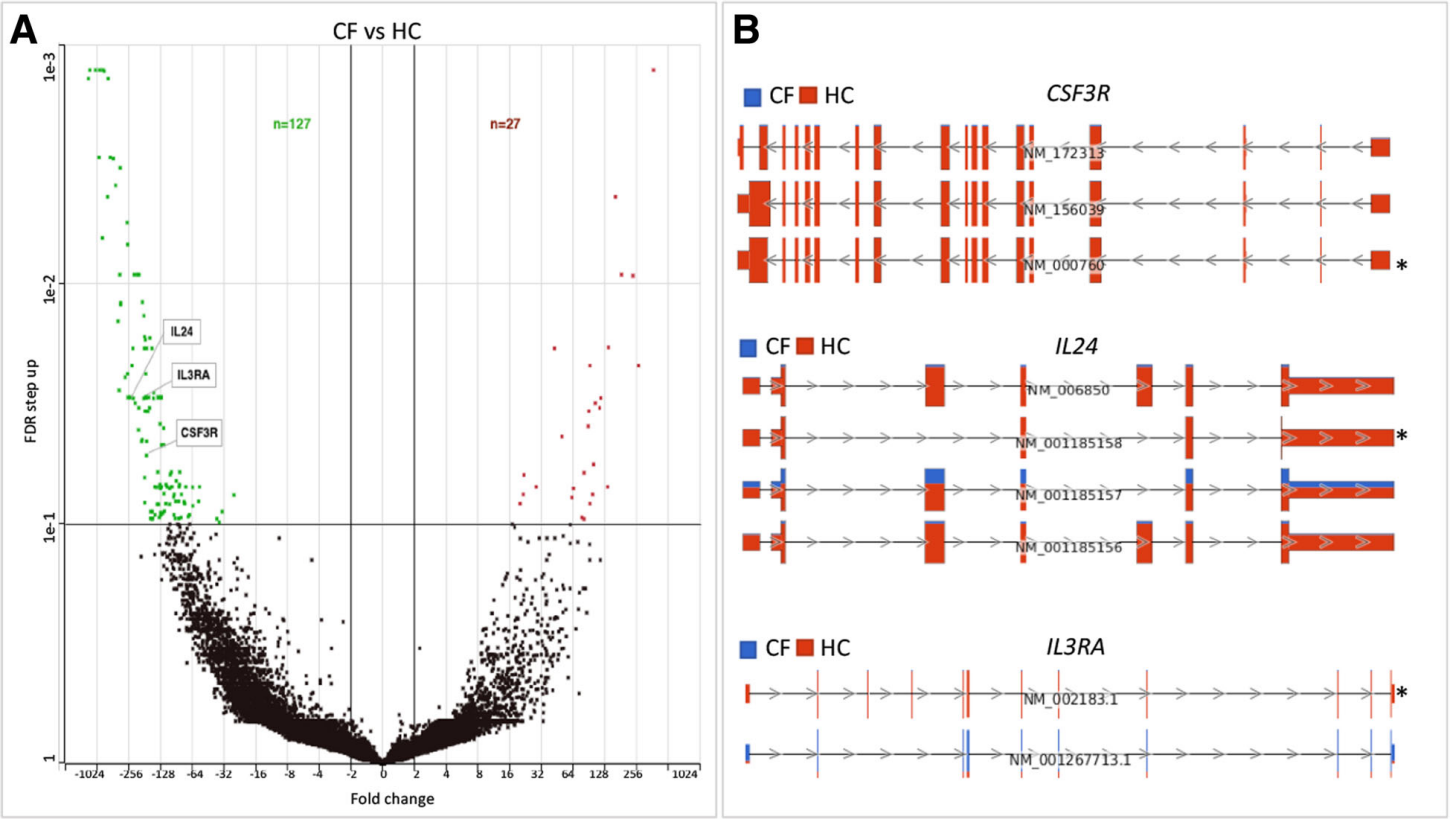
Transcript variants expression levels differ based on CF disease. a Volcano plot showing a unique set of transcript variants differentially expressed in CF versus HC samples. The upregulated and downregulated transcript variants are represented by the red and green dots respectively while those not meeting the cutoff criteria are represented by black dots. Representative dysregulated transcript variants from three genes (*CSF3R, IL24, and IL3RA*) involved in immune pathways are highlighted. **b** Genome view of transcript variants in *CSF3R* (NM_000760), *IL24* (NM_001185157), and *IL3RA* (NM_002183.1) that were differentially expressed

**Fig. 5:**
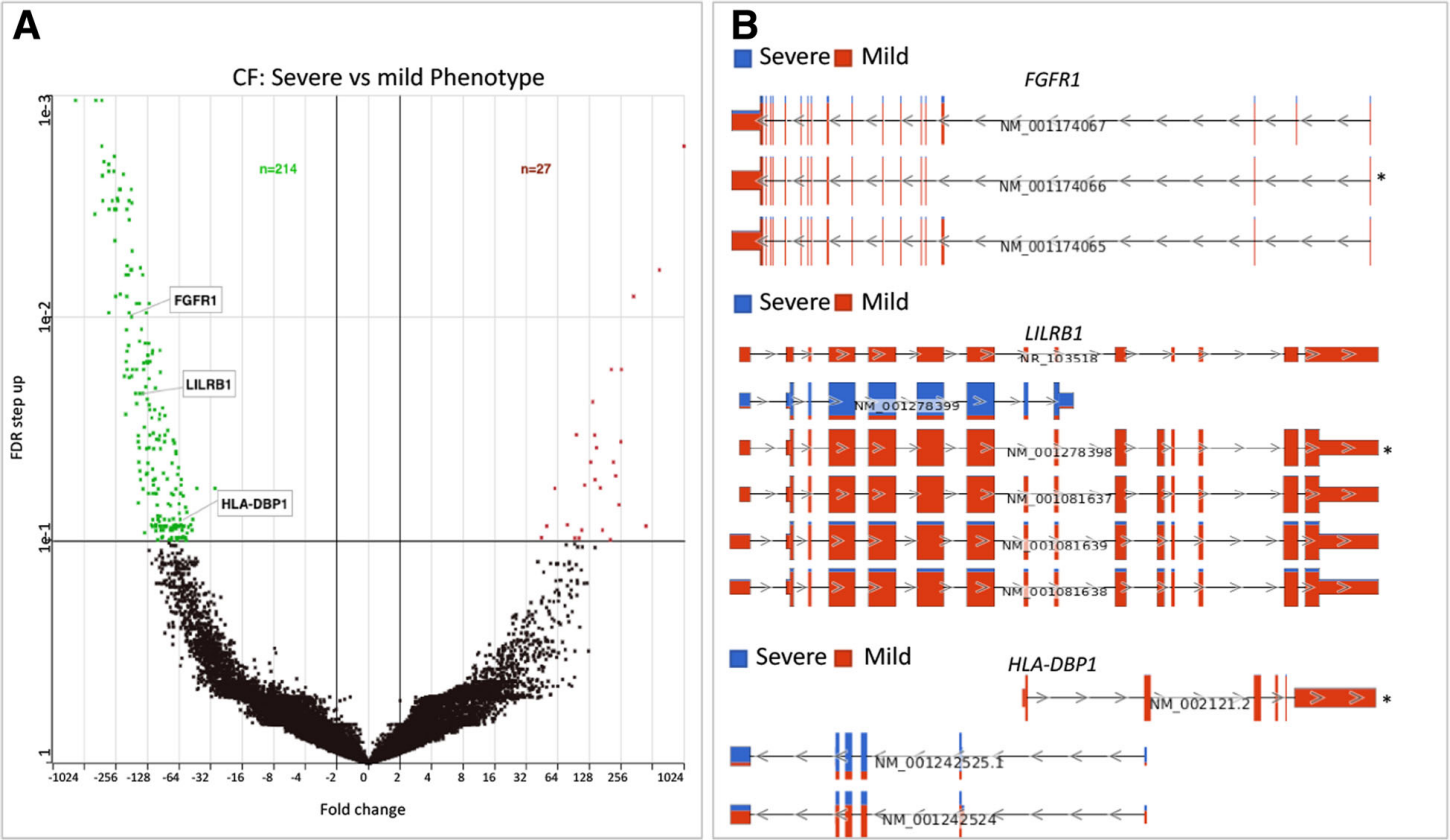
Transcript variants expression levels differ in CF based on phenotype. **a** Volcano plot showing a unique set of transcript variants differentially expressed due to CF phenotypes. The upregulated and downregulated transcript variants are represented by the red and green dots, respectively, while those not meeting the cutoff criteria are in black. Representative dysregulated transcript variants from three genes (*LILRB1, FGFR1, and HLA-DBP1*) involved in immune pathways are highlighted. **b** Genome view of transcript variants in *FGFR1* (NM_001174066), *LILRB1* (NM_001081637), and *HLA-DBP1* (NM_002121.2) that were differentially expressed

We characterized the biotype of the 32,399 RefSeq transcripts annotated in the expression dataset and further performed differential analyses to identify individual transcript variants that differ based on CF and its severe phenotype. The majority of these transcripts were protein-coding (78%), resulting from multiple-transcript genes of high abundance in the dataset (Fig. 2 b). We found 154 differentially expressed transcripts between CF and HC subjects (Fig. 4 a, Additional file 1: Table S4). These corresponded to 142 genes with varying functions, including some relevant in cytokine signaling pathway of the immune system (Colony-stimulating factor 3 receptor [*CSF3R*]*,* interleukin 24 [*IL24*]*,* Interleukin 3 Receptor Subunit Alpha *[IL3RA*] (Fig. 4 b). Also, we identified 241 transcript variants differentially expressed based on CF phenotypes. The majority (89%) of the transcript variants were downregulated in the Severe versus Mild disease group. These transcripts corresponded to 212 genes with various functions (Fig. 5 a, Additional file 1: Table S5). Interestingly, some of the genes with dysregulated transcript variants, including *FGFR1*, *LILRB1,* and *HLA-DBP1,* are also associated with various immune processes (Fig. 5 b).

### Functional analysis of differentially expressed genes

**Fig. 6:**
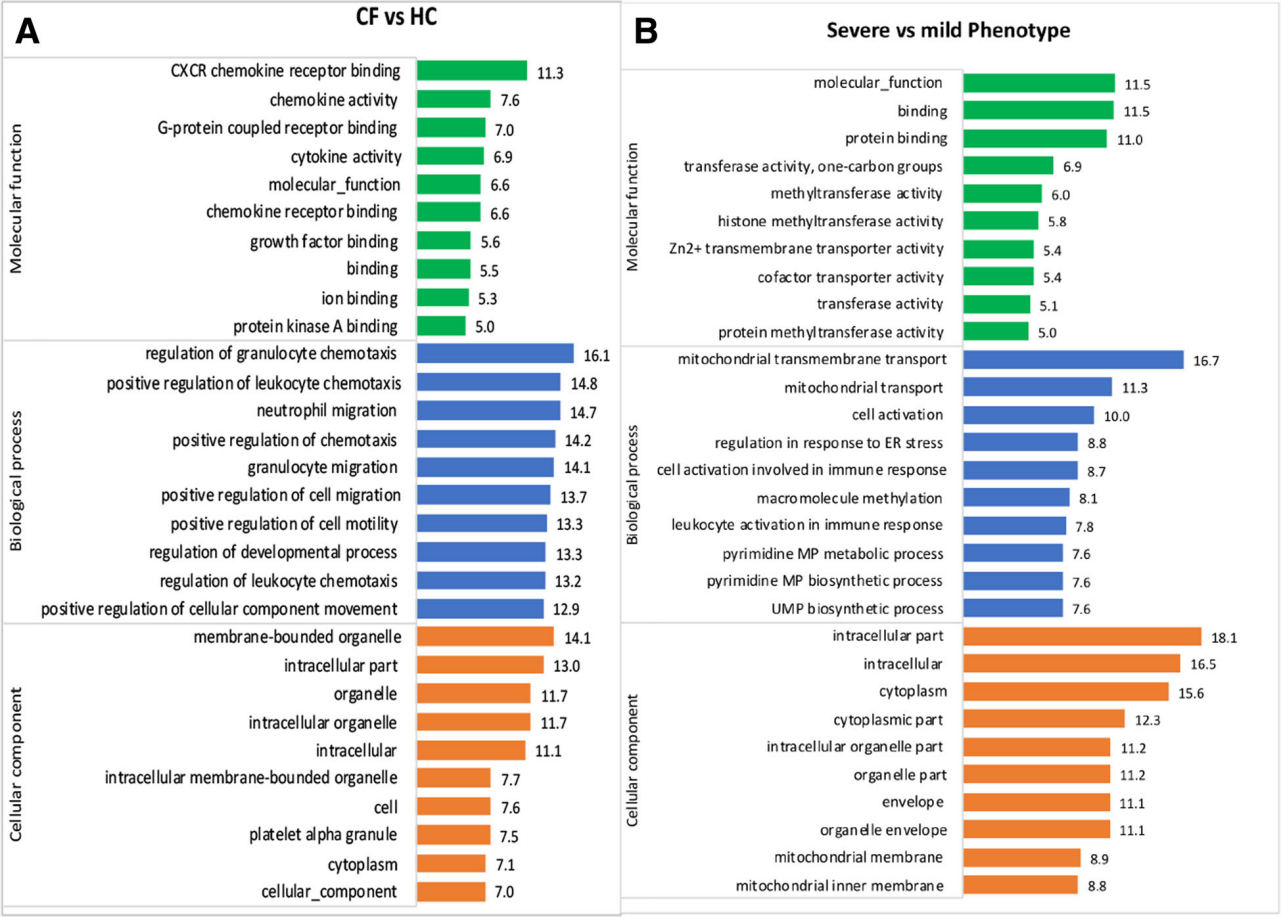
Several GO terms were associated with CF and its phenotype. **a** GO analysis performed using 151 genes dysregulated in CF versus HC samples and (**b**) GO analysis performed using 285 dysregulated genes associated with Severe versus Mild CF phenotypes. The top 10 enriched GO terms for each of the three categories (molecular function, biological process, and cellular component) are represented in the bar graphs. The negative natural logarithm of the *p*-value was used to deduce the enrichment scores shown in the bars

GO enrichment analysis performed using the significantly differentially expressed genes revealed enrichment of several GO terms in each of the three categories. Figure 6 a presents the top 10 enriched terms in each category influenced by CF-associated genes. Among the top 10 molecular function (MF) terms influenced by the dysregulated genes for this group are CXCR chemokine receptor binding, chemokine activity, G-protein coupled receptor binding, cytokine activity, and chemokine receptor. Biological process (BP) analysis showed the top 10 enriched terms were mostly associated with regulation and movement of immune cells (regulation of granulocyte chemotaxis, regulation of leukocyte chemotaxis, neutrophil migration, granulocyte migration), as well as regulation of developmental processes. The cellular component (CC) analysis showed the proteins encoded by the dysregulated genes were enriched in several cellular compartments including the membrane-bounded organelle, cytoplasm, and intracellular part (Fig. 6 a). Further enrichment analyses were performed using the gene set associated with CF phenotypes (Fig. 6 b). The MF analysis in this group indicated that the proteins encoded by the dysregulated genes were mainly associated with the MF, protein binding, transferase, and transmembrane activity terms. The top 10 enriched BP terms were mainly associated with cellular transportation and cell activation involved in immune response while CC analysis showed the proteins encoded by the dysregulated genes were also enriched in several cellular compartments including cytoplasmic, intracellular, and organelle part (Fig. 6 b).

**Fig. 7:**
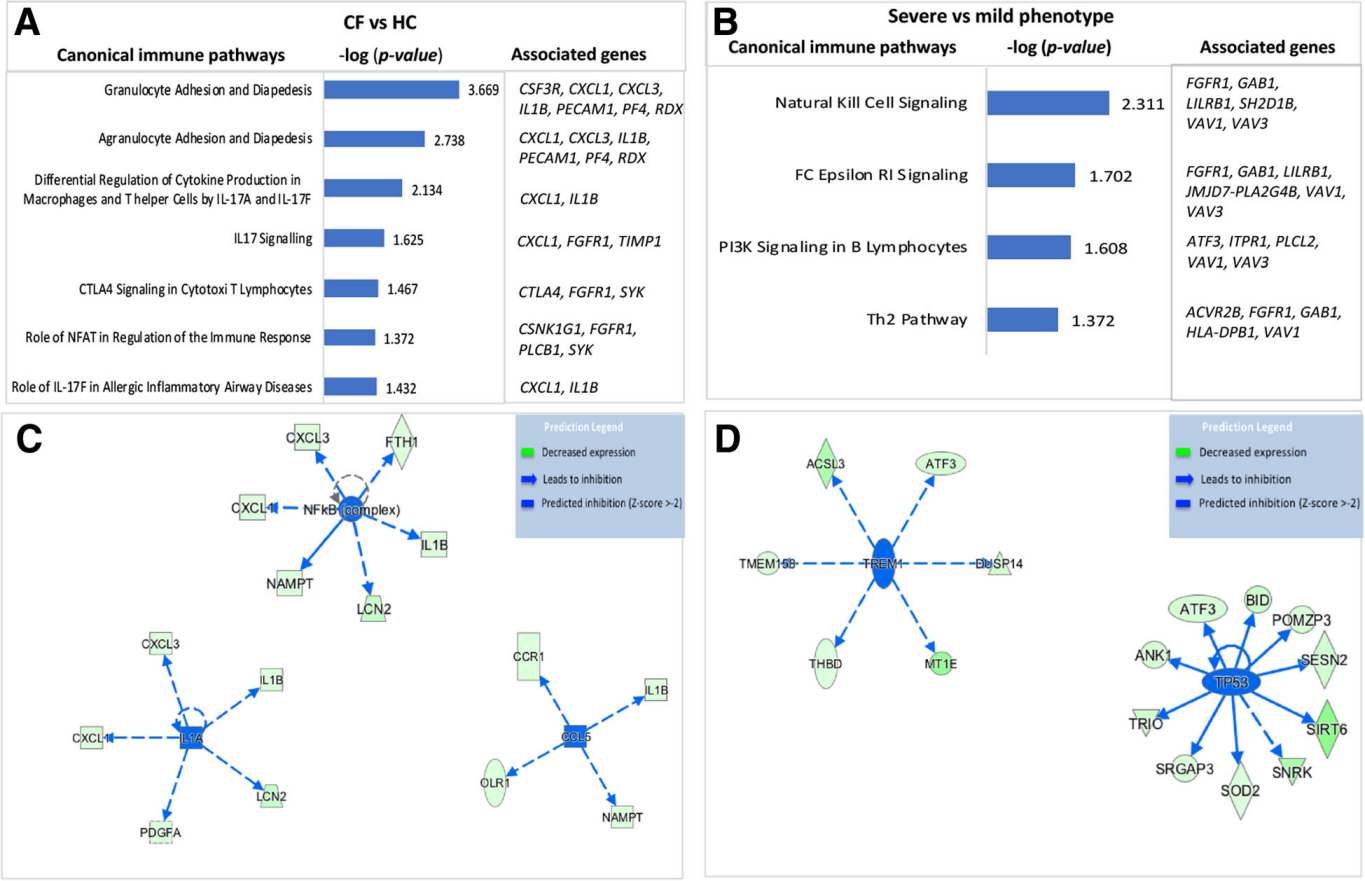
Dysregulated canonical immune pathways and upstream regulators characterized CF and its phenotype. **a** & **b** Bar graphs represent canonical immune pathways significantly associated (*p* < 0.05) with dysregulated genes in the experimental gene expression dataset. **a** Seven canonical immune pathways indicated to be dysregulated in CF when compared to HC and their associated genes (**b**) Four canonical immune pathways indicated to be dysregulated in CF based on severe versus mild phenotypes. **c** & **d** Key upstream regulators predicted to be inhibited. Three upstream regulators were predicted to be inhibited in CF compared to HC based on their associated dysregulated molecules (**c**). Two upstream regulators inhibited in CF based on phenotype (**d**)

Canonical pathway analyses identified several pathways in the IPA knowledge base that were significantly altered by the input dysregulated gene expression datasets. The immune pathways influenced by CF-associated input genes included agranulocyte/granulocyte adhesion and diapedesis, differential regulation of cytokine production in macrophages and T-helper cells by IL17A/F and IL17 signaling, and the role of IL-17F in allergic inflammatory airway disease (Fig. 7 a), while the immune pathways influenced by CF phenotypes were natural killer cell signaling, FC Epsilon R1 signaling, PI3K signaling in B lymphocytes, and the Th2 pathway (Fig. 7 b).

The upstream regulator analyses identified several upstream regulators significantly (*p* < 0.05) associated with the input gene expression dataset. Among those predicted to be inhibited in the input CF-associated gene expression dataset were Interleukin 1 Alpha (IL1A), Nuclear Factor kappa B (NF-κB) complex, and C-C Motif Chemokine Ligand 5 (CCL5), while two inhibited upstream regulators (Triggering Receptor Expressed on Myeloid cells 1 [TREM1] and Tumor protein 53 [TP53]) were associated with CF phenotype. These regulators and their associated genes in the dataset are displayed as networks (Fig. 7 c and d).

### Differentially expressed miRNAs and their interaction with molecules of immune pathways

**Fig. 8:**
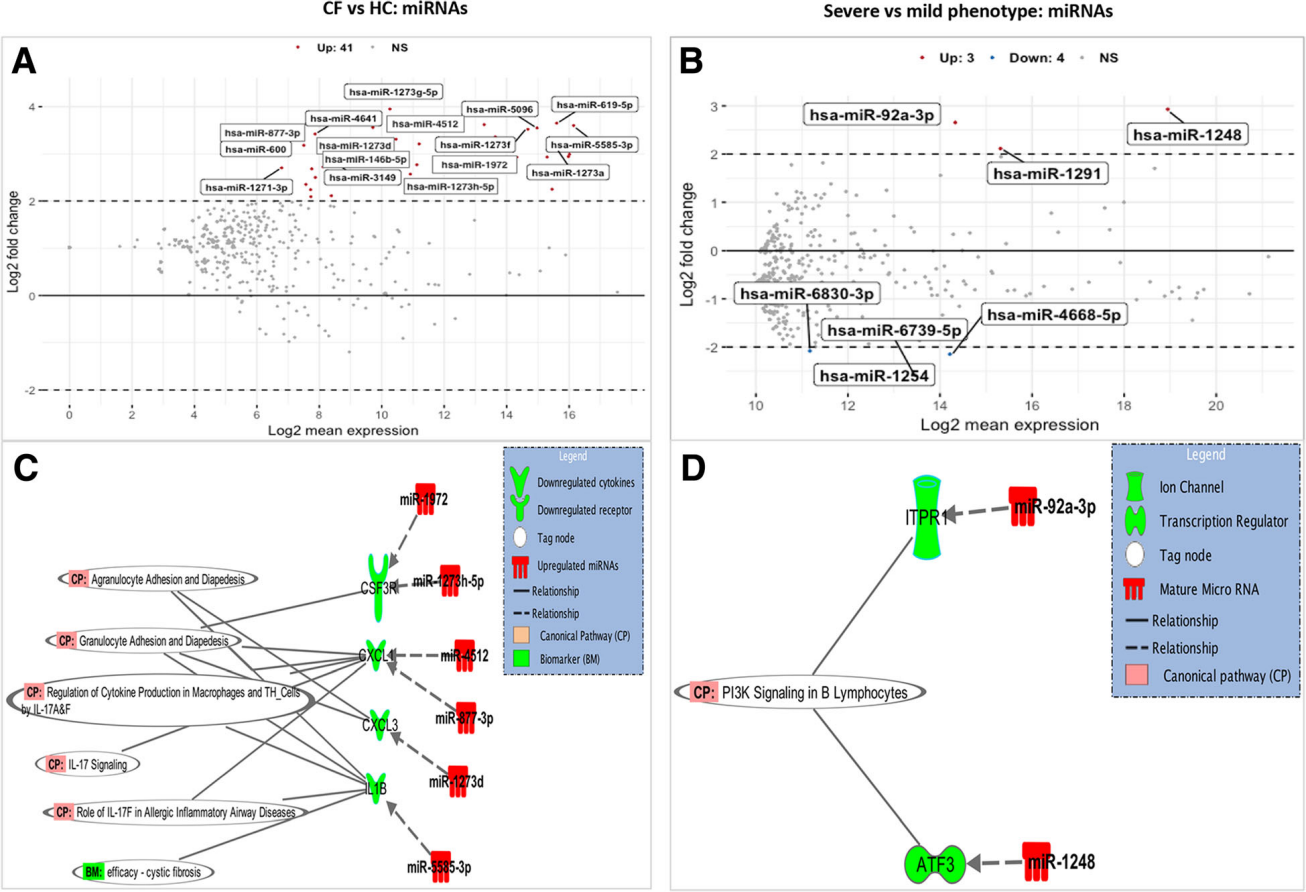
Dysregulated miRNAs in CF inversely interact with relevant dysregulated immune system mediators. **a** & **b** MA-plot segregates dysregulated miRNAs in CF compared to HC (**a**) and in CF patients with severe and mild phenotype. **b** FC values (Log2-transformed) plotted against the mean expression values (Log2-transformed) for each signature. Upregulated miRNAs are represented in red, downregulated in blue, and those not meeting the cutoff criteria (ANOVA F-test, FDR < 0.05, |log2 FC| ≥ 2) in black. **c** & **d** Inverse expression correlation of miRNAs and dysregulated genes involved in significant dysregulated canonical immune pathways in CF (see Fig. 7) (**c**) Four genes (*CSF3R*, *CXCL1*, *CXCL3,* and *IL1B*) downregulated in CF compared to HC and involved in dysregulated pathways showed inverse expression patterns with six miRNAs (miR-1972, miR-1273 h-5p, miR-4512, miR-877-3p, miR-5585-3p, miR-1273d). **d** Two dysregulated genes (*ATF3* and *ITPR1*) associated CF phenotype, and involved in a dysregulated immune pathway, showed inverse expression patterns with two miRNAs (miR-92a-3p and miR-1248)

Differential miRNA expression analysis showed 41 miRNAs (Additional file 1: Table S6) were significantly differentially expressed in CF versus HC and seven miRNAs (Additional file 1: Table S7) in Severe versus Mild CF phenotypes (ANOVA F-test, FDR < 0.05, log2 FC ≥ 2). The results are presented in MA-plots with top differentially expressed features highlighted in both the CF versus HC (Fig. 8 a) and Severe versus Mild CF comparisons (Fig. 8 b). Among the top differentially expressed miRNAs due to CF were six miRNAs that showed inverse expression patterns with their mRNA targets relevant to dysregulated canonical immune pathways reported in this study (Fig. 7 a). For example, six miRNAs (miR-1972, miR-1273 h-5p, miR-4512, miR-877-3p, miR-1273d, and miR-5585-3p) showed inverse expression correlation with four immunity-related target genes (*CSF3R*, *CXCL1, CXCL3, and IL1B*), which are prominent in dysregulated immune pathways associated with CF (Fig. 8 c). Among the seven miRNAs differentially expressed in Severe versus Mild CF phenotypes, two (miR-92-3p and miR-1248) were shown to have inverse expression correlation with two dysregulated mRNA targets (*ITPR1* and *ATF3*) involved in the PI3K signaling pathway (Fig. 8 d).

## Discussion

Impaired immune responses are a dominant feature in CF and may be central to the disease severity. In this study, we simultaneously performed total RNA sequencing and miRNA sequencing of PBMCs induced by plasma from CF subjects and HC to identify dysregulated signatures that may be involved with impaired immune responses in CF. We identified several CF-associated genes and transcripts as well as key miRNAs that may play a crucial role in modulating immune processes in CF.

Overall, we found the number of downregulated differentially expressed genes to be higher in CF and its severe phenotype (93 and 94%, respectively) compared to HC and CF mild phenotype (Fig. 3). Similarly, a higher number of downregulated genes in severe CF lung disease has been previously reported [16, 19]. This observation indicates that CF plasma induced an overall downregulatory effect on the transcriptional machinery of the wild-type PBMC compared to HC plasma. Among the top abundant genes differentially expressed due to CF were *CCR1, CSF3R*, *CXCL1*, *CXCL3*, and *IL1B* (Fig. 7 a), while those associated with CF phenotype included *ATF3, FGFR1*, *HLA-DBP1*, *ITPR1,* and *LILRB1* (Fig. 7 b). Together, the overall enrichment analysis in this study indicated many dysregulated genes associated with CF and its phenotype are involved in several immune signaling pathways and enriched in several cellular compartments (Fig. 6). Hence, our results support previous findings that expression signatures of PBMCs differ at the gene level in CF and several immunity-related genes are dysregulated [19]. However, we further characterized biological mechanisms that may underlie gene expression differences by analyzing differential expression at the transcript level.

As most human genes have multiple transcript variants generated by alternative splicing events that can be disease-specific and may underlie gene expression differences [24], we explored if individual transcripts characterized CF and its phenotypes. Indeed, we identified several transcript variants of multiple-transcript genes associated with CF and its phenotypes. Among those associated with CF were transcript variants from genes encoding cytokines and cytokine receptors including *CSF3R*, *IL24,* and *IL3RA*. Alternative splicing tailors the activity of cytokines and their receptors to specific pathological conditions, for example, by creating isoforms with antagonistic effects [25, 48]. Thus, alternative splicing is a major regulatory mechanism through which cytokine signaling can be altered. Since differential expression of cytokines is common in CF [11, 12, 49], our results support the notion that alternative splicing may contribute to cytokine imbalances in CF. Additionally, we identified many individual transcripts differentially expressed due to CF phenotype were encoded by genes involved in several corresponding dysregulated immune pathways, including transcript variants of *FGFR1*, *LILRB1*, and *HLA-DBP1* (Fig. 5). Many signaling pathways are altered in cancer due to expression changes in transcript variants of genes relevant in these pathways [50]. Thus, our findings shed new insights into a molecular mechanism that may be potentially involved in impaired immune processes in CF. Further study using more coverage depth is encouraged to delineate a comprehensive impact of alternative splicing to the transcriptome diversity of CF immune cells.

Further in silico predictions using IPA implicated some inhibited upstream regulators that may be responsible for the underlying differences in the expression of immunity-related genes. Three key transcriptional regulators (CCL5, IL1A, and NF-κB) featured among the top regulators predicted to be inhibited due to CF (Fig. 7 c). The protein encoded by *IL1A* is a cytokine belonging to the IL-1 family that plays various important roles in immune processes and hematopoiesis. IL1A regulator was predicted to be inhibited based on the downregulation of its targets including *IL-1B*, a reported CF modifier gene [51]. Also, the inhibited CCL5 plays an essential role during inflammation by inducing the migration of blood leukocytes to sites of infection in order to initiate immune responses against invading pathogens [52]. Based on its critical role in the immune system, several studies have investigated the transcription factors that may be involved in its regulation [53, 54]. One such factor, NF-κB, was reported to mediate the transcription and production of CCL5 [54], and was also predicted in this study to be inhibited due to the downregulation of its receptor (*CCR1*) and other relevant target genes (Fig. 7 c). Also, NF-κB inhibition has been demonstrated to result in reduced expression of CCL5 and cell survival factors released by PBMCs [55]. Thus, our findings suggest that dysregulated NF-κB regulators leading to reduced CCL5 expression may result in reduced ability to recruit PBMCs in CF. Further, NF-κB induces the transcription of several genes in the immune system that are important for regulating inflammation, chemotaxis, and antimicrobial activity [56]. Although NF-κB activation leading to increased expression of proinflammatory cytokines in CF epithelial cells has been reported [57–59], our analysis predicted inhibition of NF-κB regulator due to the downregulation of its target genes in CF plasma-induced PBMCs (Fig. 7 c). Given that previous studies using PBMCs have shown NF-κB has an inhibitory effect on immunity-related genes when suppressed [60, 61], it is conceivable that NF-κB is downregulated as predicted.

A major upstream regulator predicted to be impaired due to CF phenotype (Fig. 7 d) is TREM1, which is known to play a crucial role in immune responses triggered by bacterial products via activation of circulating neutrophils and monocytes. A previous study that examined TREM1 in circulating monocytes of CF patients reported a downregulation of TREM1 compared to healthy subjects [62]. CF patients with severe disease have an increased risk of chronic bacterial infection compared to those with the milder disease [41]. Our findings indicate lower levels of TREM1 in the Severe CF group, leading to the downregulation of immunity-related genes, may account for increased susceptibility to bacterial infection in severe CF. Also associated with CF phenotype was TP53, which encodes the p53 protein, the main functions of which are to respond to cellular stress, regulate cell division, and initiate DNA repair. Although there is limited literature on the role of TP53 in CF immune cells, it is a molecular target in several cancer studies due to its role as a tumor suppressor [63]. The mechanistic activation of p53 and NF-κB are similar and both can co-regulate the expression of many inflammatory genes including cytokines and chemokines [64]. Although p53 and NF-κB are expected to play opposing roles in cellular response to stress, they have also been shown to work in the same direction [65, 66]. Also, in a recent study utilizing two models (circulating tumor cells and PBMCs) for gene expression profiling in breast cancer, *TP53* was upregulated in the tumor cells but downregulated in PBMCs [67]. These findings suggest the regulatory role of p53 may be cell-type- or disease-specific. However, our results indicate dysregulation of *TP53* in the Severe CF group may be attributable to impaired immune processes.

Further immunological consequences of the dysregulated genes were elucidated via canonical pathway analysis. The pathways influenced by CF included agranulocyte/granulocyte adhesion and diapedesis, differential regulation of cytokine production in macrophages and T helper cells by IL-17A and IL-17F, and role of IL-17F in allergic inflammatory airway diseases, while the immune pathways influenced by CF phenotype were natural killer cell signaling, FC Epsilon R1 signaling, PI3K signaling in B lymphocytes, and Th2 pathway. Several differentially expressed genes featured prominently in the dysregulated pathways (Fig. 7 a & b).

miRNAs have gained significant attention for their role in several biological and pathological processes by suppressing the expression of their target genes [68], including *CFTR* in CF [69]. We investigated differentially expressed miRNAs induced by CF and its phenotypes, then analyzed their association with the corresponding genes involved in the dysregulated immune pathways. While several studies have reported miRNAs dysregulation in CF epithelial cells [28, 29], there is limited literature about dysregulated miRNAs in CF PBMCs. We identified 41 differentially expressed miRNAs associated with CF and 7 associated with CF phenotypes. These miRNAs were targets for various genes in the corresponding gene expression dataset, including several immunity-related genes. Among these, we identified six miRNAs (miR-95-5p, miR-4512, miR-877-3p, miR-1273d and miR-5585-3p) with inverse expression patterns to four prominent genes (*CSF3R*, *CXCL1*, *CXCL3*, and *IL1B*) within key impaired immune pathways identified due to CF (Fig. 8 c). Interestingly, both the chemokines (*CXCL1* and *CXCL3*) and cytokine (*IL1B*) are known to play major roles during inflammation and have been associated with CF immune responses in several studies [70, 71]. The identification of dysregulated miRNAs interacting with these genes suggests that miRNAs may contribute to the underlying gene expression differences in CF and further implicates these molecules as potential players involved with impaired immune pathways.

Further, two upregulated miRNAs (miR-92a-3p and miR-1248) targeted two downregulated genes (*ITPR1* and *ATF3*, respectively) that feature prominently in the dysregulated PI3K signaling pathway associated with CF phenotypes (Fig. 8 d). In the immune system, an impaired PI3K signaling pathway is associated with immunodeficiency, while unrestrained PI3K signaling is associated with autoimmunity. While literature on miR-92a-3p expression levels in CF PBMCs is still lacking, there have been several reports of elevated levels of mir-92a-3p in numerous cancers [72–74]. The miR-92a family of miRNAs are known for playing an essential role in regulating the development of vital organs in the cardiovascular and pulmonary system [75]. Intriguingly, PI3K signaling has been demonstrated as the downstream pathway for miR-92a-3p in cancer cells [76]. Thus, supporting the results of the in silico analyses in this study, miR-92a-3p may have a regulatory role in the observed impaired PI3K signaling pathway associated with CF phenotype. In addition, increased expression levels of miR-1248, which was associated with more severe disease in our study, has been reported in many diseases including asthma [77], adenovirus infection [78], and cancer [79]. We predicted further using IPA that miR-1248 was inversely correlated with a major dysregulated gene (*ATF3*) associated with CF phenotype and thus the impairment of the PI3K signaling pathway. Thus, future work may investigate if modulating the expression of these miRNAs alters the expression of their targets or yields therapeutic benefits. Taken together, our results suggest that miRNAs are potential transcriptional and network regulators in CF involved with impaired immune responses.

Confirmatory studies focusing on selected targets identified in this study are encouraged for validation and to potentially unravel novel molecular drivers that hold promise as targets for CF therapeutics.

## Conclusions

We utilized RNA-Seq in this study to identify genes, transcripts, and miRNAs in stimulated PBMCs that characterize CF and its phenotypes. We conclude that CF plasma induced an overall downregulatory effect on the transcriptional machinery of wild-type PBMC compared to HC plasma. Although many immune response genes were downregulated, the CF immune response is usually exacerbated. Thus, the observation in PBMCs induced with CF plasma is possibly influenced by complex regulatory mechanisms within the immune system, which warrants further characterization. Our findings support previous array-based studies by demonstrating that gene expression signatures in PBMCs differ due to CF and can distinguish between severe and mild phenotypes. In addition, we demonstrated for the first time using plasma-induced PBMCs that unique transcript variants from multiple-transcript genes differ in expression, distinguish CF and its phenotypes, may underlie the observed gene expression differences, and are involved with impaired immune pathways in CF. Lastly, we identified miRNAs as potential transcriptional and network regulators associated with impaired immune responses in CF. Future work is needed to validate the molecular targets identified in this study and explore their therapeutic potential for CF using larger sample sizes.

## Additional file

Additional file 1: **Table S1.** Total RNA-Seq summary statistics. **Table S2.** List of differentially expressed genes in CF vs HC. **Table S3.** List of differentially expressed genes in Severe vs Mild CF. **Table S4.** List of differentially expressed transcripts in CF vs HC. **Table S5.** List of differentially expressed transcripts in Severe vs Mild CF. **Table S6.** List of differentially expressed miRNAs in CF vs HC. **Table S7**. List of differentially expressed miRNAs in Severe vs Mild CF. **Table S8**. Sample information. (XLSX 151 kb)

## Abbreviations

ANOVA: Analysis of variance
ATF3: Activating Transcription Factor 3
BP: Biological process
CC: Cellular component
CCL5: C-C Motif Chemokine Ligand 5
CCR1: C-C Motif Chemokine Receptor 1
cDNA: Complementary DNA
CF: Cystic fibrosis
CFTR: Cystic fibrosis transmembrane conductance regulator
CSF3R: Colony-stimulating factor 3 receptor
CXCL1: Chemokine (C-X-C motif) ligand 1
CXCL3: Chemokine (C-X-C motif) ligand 3
CXCR: Chemokine (C-X-C motif) receptor
E/M: Expectation/Maximization
EDTA: Ethylenediaminetetraacetic acid
FDR: False discovery rate
FEV_1_: Forced expiratory volume in 1 s
FGFR1: Fibroblast growth factor receptor 1
FTH1: Ferritin Heavy Chain 1
GO: Gene ontology
HC: Healthy controls
HLA: Human leukocyte antigen
HLA-DBP1: Major histocompatibility complex, class II, DP beta 1
IL17: Interleukin 17
IL1A: Interleukin 1 Alpha
IL1B: Interleukin 1 Beta
IL24: Interleukin 24
IL3RA: Interleukin 3 Receptor Subunit Alpha
IPA: Ingenuity pathway analysis
IQR: Interquartile range
IRB: Institutional review board
ITPR1: Inositol 1,4,5-trisphosphate receptor type 1
LILRB1: Leukocyte immunoglobulin like receptor B1
MF: Molecular function
miRNAs: Micro RNAs
mRNAs: messenger RNAs
NF-κB: Nuclear Factor kappa B
Pa: *Pseudomonas aeruginosa*
PBMCs: Peripheral blood mononuclear cells
PCA: Principal component analysis
PCR: Polymerase chain reaction
PI: Pancreatic insufficient
PI3K: Phosphoinositide-3-Kinases
PS: Pancreatic sufficient
RNA-Seq: RNA Sequencing
rRNA: Ribosomal RNA
Th2: T helper 2
TMM: Trimmed mean of M values
TP53: Tumor protein 53
TREM1: Triggering receptor expressed on myeloid cells 1
